# A card game for the treatment of delusional ideas: A naturalistic pilot trial

**DOI:** 10.1186/1471-244X-6-48

**Published:** 2006-10-30

**Authors:** Yasser Khazaal, Jérôme Favrod, Joël Libbrecht, Sophie Claude Finot, Silke Azoulay, Laetitia Benzakin, Myriam Oury-Delamotte, Christian Follack, Valentino Pomini

**Affiliations:** 1 Département de Psychiatrie, Echallens 9, CH-1004 Lausanne, Switzerland; 2 Département de psychiatrie – Site de Cery – CH-1008 Prilly, Switzerland; 3 Les Marronniers, Rue Despars 94, B-7500 Tournai, Belgium; 4 Hôpital psychiatrique de Malévoz, CH-1870 Monthey, Switzerland; 5 Centre Psychosocial, Chante-Merle 84, CH-2502 Biel, Switzerland; 6 Hôpital Chalucet, F- 83000 Toulon, France; 7 Hôpital St-Jean de Dieu, F-69008 Lyon, France; 8 Hôpital psychiatrique de Marsens, CH-1633 Marsens, Switzerland

## Abstract

**Background:**

"Michael's game" is a card game which aims at familiarizing healthcare professionals and patients with cognitive behavioral therapy of psychotic symptoms. This naturalistic study tests the feasibility and the impact of the intervention in various naturalistic settings.

**Method:**

Fifty five patients were recruited in seven centers. They were assessed in pre and post-test with the Peters Delusion Inventory – 21 items (PDI-21).

**Results:**

Forty five patients completed the intervention significantly reducing their conviction and preoccupation scores on the PDI-21.

**Conclusion:**

This pilot study supports the feasibility and effectiveness of "Michael's game" in naturalistic setting. Additional studies could validate the game in a controlled fashion.

## Background

Cognitive and Behavioural therapies (CBTs) of psychotic symptoms have been developed [1] with the aim to reduce the distress associated with delusional ideas and hallucinations and also to improve the patients' coping ability. These approaches have been studied as complements to pharmaceutical treatments. Recent meta-analyses demonstrate their utility for the treatment of psychotic conditions [2-5]. Even if this effect size may be considered modest [5] in those studies which add CBTs to already efficacious treatments [6], CBTs of psychotic symptoms remain a promising approach.

Indeed, even though the approach doesn't have the expected effect on the reduction of the symptoms, it enhances insight, as well as reduces the suffering and the pathological behaviour resulting from the psychotic symptoms [7-10]. The frequency of residual psychotic symptoms in patients treated with antipsychotic drugs [11], the positive outcome of cognitive therapy in this particular situation, the lack of trained personnel and the difficulties starting and maintaining treatment with some of the patients [12,13] call for the development of new approaches.

Among 100 individuals showing delusional ideas, 3/4 of them do not offer alternative explanations for their delusional ideas [14]. The lack of alternative explanation could be detailed as follows:

1) Patients can't find an alternative explanation because of the fact that it can be difficult to distinguish an internal event (hallucination) from an external one; or it may be difficult to conceive of an alternative explanation for an internal event.

2) Patients do not manage to use their ability to come up with alternatives. This could be caused by cognitive biases such as "jumping to conclusions" [14], need for closure [15], or confirmatory reasoning. These biases could prevent patients from considering alternatives.

3) Finally, alternatives could be avoided because their consequences are too stressful for the person. For example, the alternative: "I am becoming crazy" is probably unacceptable for most people and therefore, it is at risk of being rejected.

Whereas research has yet to confirm mechanism of action of CBTs of psychotic symptoms [16], it seems to help clients develop alternative explanations for their experiences by stressing the contradictions underlying these ideas, Socratic questioning and reality tests, thus allowing access to more functional alternatives [17]. Finally, CBTs tackle psychotic symptoms in a normalizing perspective, placing them on a continuum with normal experiences and bringing the understanding of psychotic themes and patients' reactions to them closer to real-life experience [18]. Finally, the aim of CBTs is for the patient to master the therapeutic process of questioning the delusional ideas [19].

Most of the studies evaluating CBTs of psychotic symptoms have been efficacy studies, where treatments were delivered by teams of specialized and/or supervised professionals [19]. Few studies have assessed the effectiveness of CBTs of psychotic symptoms in routine clinical practice. However recent studies are conclusive about their utility within a multidisciplinary psychiatric setting with therapists qualified in CBTs, or therapists who are very experienced in the clinic of psychotic symptoms [17] as well as community psychiatric nurses trained to deliver brief CBT intervention [20,21].

This study aims at assessing a game designed to make its users accustomed to CBTs of psychotic symptoms and was run in various naturalistic settings, with therapists who have different levels of training in CBTs.

## Methods

### Intervention

"Michael's Game", a training module for hypothetical reasoning is a treatment inspired by CBTs of psychotic symptoms. It was conceived by the first two authors as a tool to promote the dissemination of CBTs in natural clinical settings. Principles of the game are founded on cognitive therapy of psychotic symptoms [22,23] and use their techniques [24] such as: developing a therapeutic alliance based on the patient's perspective, normalizing psychotic symptoms, cognitive restructuring techniques aiming to develop alternative explanations to their delusions, reality testing and connecting belief to emotion and behavior. It could be used as a preliminary or complement of individual CBTs. "Michael's game" is a program aiming at training hypothetical reasoning. Participants have to help Michael to find alternatives to the erroneous conclusions that Michael draws from situations described on each card. It was conceived as a group card game in order to allow patients to become partners of a fictive character (Michael) interacting together with cards containing impersonal information which may however reflect their own problems. The game was introduced to patients by asking them if they would like to participate in a study evaluating a game, called "Michael's game", which requires and trains hypothetical reasoning.

Each card number corresponds to a situation and objectives that target, through progressive stages, reasoning with hypotheses. One or two caregivers lead the game. The sessions last for 60 to 90 minutes, once a week. The participants are led through specific questions the participants are led to correct the erroneous conclusions that Michael draws from situations he is confronted with. The game was conceived according to two axes of progression:

- The kind of situations presented (non-psychotic, non-emotional: cards 1–11), (emotional, non-psychotic: cards 12–32) then (psychotic cards: 33–79) (Table 1).

**Table 1:** Examples of cards

**A non-psychotic and non-emotional situation card:**
Michael sets two bags of different sizes on each side of a scale.
The big bag has the same weight as the small bag.
Michael is surprised since the two bags are supposed to be filled with cotton.
He thinks that the small bag contains a stone.

**A psychotic situation card:**
Michael is watching his favorite show on television.
When the show host appears, Michael is so pleased that he bursts out laughing.
The show host and another participant in the show start laughing at the same time.
Michael tells himself: "My joy is catching them".

- The progression of the objectives of each card (identifying the situation, Michael's hypothesis, arguments for and against a hypothesis, emotional and behavioural consequences of the hypothesis, etc.) (Table 2).

**Table 2:** Examples of the objectives of the cards

- Describe a situation before interpretation
- Devise the interpretation of a situation as a hypothesis
- Search for different interpretations of the same situation
- Identify the cognitive and behavioural consequences of the different hypotheses
- Search for a link between the interpretation given for a situation and a personal real-life experience
- Put the hypotheses in hierarchical order in terms of their probability
- Search for arguments for or against a hypothesis
- Conceive a way of testing a given hypothesis in reality

Cards are addressed one after the other and therefore the timing of the progression between the different stages of the intervention (non emotional, emotional, psychotic situations) varies as it is determined by the time discussions take.

### Subjects

A sample size of 32 patients was sufficient to provide 80% power to detect a statistically significant reduction of 20 points in PDI scores with a type I error of 0.05. The study included 55 patients, 45 at endpoint which is higher than the minimum indicated by the sample size estimation.

Fifty five subjects were recruited in cohorts of 3 to 7 patients. To be included in the study patients had to be stabilized on anti-psychotic medication and still present psychotic symptoms. Thirty three patients were recruited in Switzerland from state psychiatric hospitals in Malevoz and Marsens and outpatient community centres in Bienne and in Lausanne. Thirteen patients were selected from two hospital units in Tournai, Belgium, one of which being a forensic unit, and 9 from outpatient clinics in Toulon and Lyon, in France. The average age of the sample is 27 years (SD 5.7). It consists of 18 women and 37 men. Diagnoses are established by experienced clinicians from the psychiatric services teams and were drawn from medical records. The DSM-IV diagnoses are divided up into 43 schizophrenia, 8 schizo-affective disorders and 4 depressions with psychotic characteristics. With regard to accommodation: 40 are living in independent apartments, 9 in nursing homes and 6 have been staying in a forensic hospital for more than 6 months.

### Game leaders

The game leaders are 9 nurses (5 having no prior CBTs training, and 4 having some minimal previous training) and 3 psychologists (with some minimal previous training in CBTs). These professionals are not expected to be already trained in cognitive therapy. The game leaders received a two hour long introduction to "Michael's game" either in a dedicated workshop (Malévoz, Marsens, Lausanne, Bienne), or during the 2 to 3 day CBT training course (Tournai, Lyon, Toulon). To ensue that therapists delivered the intervention as intended, the game leaders were trained by one of the first two authors. The workshop comprised a brief presentation of CBTs of psychotic symptoms rationale and technique followed by a description of the game and one hour role plays. Supervision was provided during the study by various means including telephone, email, group or individual sessions with one of the game authors.

### Measures

The Peters & al. Delusion Inventory 21 items (PDI-21) [25] was administered in pre- and post-tests. The PDI-21 is a self-report questionnaire, which assesses the presence of 21 beliefs, expressed in common language. When the patient checks the presence of a symptom, s/he has to fill in three 5-point Likert scales which measure the intensity of anxiety, preoccupation and conviction associated with the symptoms. The PDI-21 distinguishes psychotic patients from healthy subjects [26] and shows a good correlation with the Brief Psychiatric Rating Scale (BPRS) in patients who have received a diagnosis of schizophrenia, but who are stable on a clinical level [27]. The Cronbach alpha coefficient of the original version of the PDI-21 is 0.82, indicating a good internal consistency of the instrument [28]. We found a Cronbach alpha of 0.835 in our sample. This result is very similar with that given by Peters and colleagues."

Participants gave informed consent and local Ethical Committee approval was obtained for the study.

## Results

Among the 55 patients who accepted to participate in the study, 10 dropped-out (7 left the group and 3 were not available at post-test due to their departure from the hospital). There are no statistical differences on clinical and demographic variables between patients who completed the study and patients who dropped-out, except for age. Patients who dropped-out were significantly younger (t = 2.29, df (53), 2-tailed p = .03) : the mean age of the sample was 33.4 years (sd = 8.4) whereas the mean age of the drop out subjects was 27.0 years (sd = 5.7). Drop out seems to be due to acute episodes (2 patients, one of them asking for his admission to a future group session), modification of treatment site (five of them continued their psychiatric treatment as planned) and was associated to complete withdrawal of outpatient treatment (3).

The average number of sessions used to direct the group in the different sites is 12.2 sessions (SD 4.9). The average level of participation is 74 % (SD 36 %).

In table 3 we report the pretest and post-test scores of the PDI-21 (number of symptoms experienced and proportional scores for anxiety, preoccupation and conviction), and the corresponding effect sizes as well. Proportional scores go from 1 to 5 and correspond to the raw scores divided by the total number of symptoms experienced by the patients. Effect sizes were calculated using Cohen's d formula, with pre-tests scores as control group comparison measures and post-tests scores as experimental group measures. The effect sizes can be considered as very low to moderate, with preoccupation and conviction as the dimensions where the major modifications were found. This is confirmed by the paired t-tests analyses showed in table 4: only the differences observed for preoccupation and conviction appear to be statistically significant.

**Table 3:** Pretest and post-tests scores

	Pretest Mean (sd)	Post-test Mean (sd)	Effect size Cohen's d
Symptoms	8.71 (5.07)	7.56 (5.16)	-.22
Anxiety	2.56 (1.23)	2.39 (1.32)	-.13
Preoccupation	2.76 (1.04)	2.14 (1.23)	-.54
Conviction	3.47 (1.18)	2.78 (1.57)	-.50

**Table 4:** Paired Sample t-tests

	Paired Differences		99% Confidence Interval				
	Mean	s.d.	Lower	Upper	t	df	2-tailed p
Symptoms	1.16	4.98	-.84	3.15	1.56	44	.127
Anxiety	.17	1.53	-.44	.78	.75	44	.459
Preoccupation	.62	1.21	.14	1.11	3.46	44	.001
Conviction	.69	1.68	-.02	1.36	2.76	44	.008

Note. A positive value of mean difference at a PDI score indicates that the patient has a smaller score at post-test, corresponding to a reduction in self-reported symptoms.

Impact of socio-demographic variables and diagnostic on outcome (in terms of mean differences between pre and post-tests PDI-21 scores) were tested through independent T-tests and (sex, diagnostic groups: schizophrenia vs others, age groups: < 33 years vs. ≥ 33 years old, independent apartments vs nursing homes or hospital settings) and ANOVA (country origin). Results of these analyses show no statistically significant differences between groups. These observations seem to indicate that these patients' characteristics do not have influence on the impact of "Michael's game".

## Discussion and conclusion

This pilot study supports the feasibility of this therapeutic approach and the ease of its diffusion in various clinical settings. "Michael's game" has been used easily, after a short training, in different sites that were not specialized in CBTs. In addition, the drop out rate (18%) is weaker than in other naturalistic studies [19], but it is may be due to the small sample size. The absence of differences noted in the results of the three participating countries indicates a good reproducibility of the intervention. The reduction of the PDI-21 scores suggests that "Michael's game" could have a therapeutic effect, although the clinical impact of these differences is difficult to establish in the absence of a more controlled design. The game seems to stimulate interest and curiosity in patients who may then experience revelations about themselves (i.e. one patient exclaimed in a discussion on Michael hearing voices, that he too experienced the same thing when observing attractive objects) or start questioning their own experience. The game could possibly be useful as an introduction or complement to an individual CBT, as a training for hypothetical reasoning, or as a stimulator easing collaborative discussions between patients and caregivers.

The intervention could probably be used on all patients with psychotic symptoms maybe with the exception of those with comorbid mental retardation or severe conceptual disorganisation

This study has several limitations such as its non-controlled nature, the use of a self-report questionnaire, the non-controlled character of the pharmacological treatment, as well as the absence of follow-up measurement. Additional studies could validate the game in a controlled fashion. Finally, its impact on individual CBT treatment, long term adhesion to outpatient treatment, or future hospitalization as well as its impact on the CBT trainings of therapists could be assessed.

## Competing interests

The author(s) declare that they have no competing interests.

## Authors' contributions

YK and JF wrote the "Michael's game". YK and JF participated in the design of the study. YK, JF and VP drafted the manuscript. VP performed the statistical analysis. JL, S-C F, L B, M O-D and C F are game leaders and participated to the recruitment and the collection of the data. All authors read and approved the final manuscript.

## Pre-publication history

The pre-publication history for this paper can be accessed here:


